# A rapid review of the barriers and facilitators of mental health service access among Veterans and their families

**DOI:** 10.3389/frhs.2024.1426202

**Published:** 2024-07-22

**Authors:** Natalie Ein, Julia Gervasio, Kate St. Cyr, Jenny J. W. Liu, Clara Baker, Anthony Nazarov, J. Don Richardson

**Affiliations:** ^1^ MacDonald Franklin OSI Research and Innovation Centre, Lawson Health Research Institute, London, ON, Canada; ^2^ Department of Psychiatry, Western University, London, ON, Canada; ^3^ Department of Psychology, Toronto Metropolitan University, Toronto, ON, Canada; ^4^ Dalla Lana School of Public Health, University of Toronto, Toronto, ON, Canada; ^5^ Department of Psychiatry and Behavioural Neurosciences, McMaster University, Hamilton, ON, Canada; ^6^ St. Joseph’s OSI Clinic, St. Joseph’s Health Care London, London, ON, Canada

**Keywords:** mental health services, Veterans, Veteran families, wellbeing, mental health

## Abstract

**Introduction:**

Transitioning to civilian life after military service can be challenging for both Veterans and their families. Accessible mental health services are crucial during this period to provide support. The objective of this review was to conduct a rapid review to capture the barriers and identify facilitators that influence access to mental health services for Veterans and their families during the post-service transition period.

**Methods:**

This review was conducted using the Cochrane Handbook for Systematic Reviews of Interventions as a methodological framework and followed the Preferred Reporting Items for Systematic Reviews and Meta-Analyses - Rapid Review (PRISMA-RR).

**Results:**

A total of 60 articles and 67 independent samples were included in the final data analyses. Across the included articles, this review identified 23 barriers and 14 facilitator themes. Issues navigating the mental health care system was identified as the main challenge among Veterans and their families, and those who received support navigating the system identified this as a significant facilitator. Applying the Theoretical Domains Framework, most of the identified barriers and facilitators were categorized into environmental context and resources domain.

**Discussion:**

The heterogeneity in Veterans' and Veteran families' experiences with mental health care-seeking may suggest that access to mental health care for Veterans and Veteran families cannot be solved by addressing one type of barrier alone. Instead, coordinated efforts to address prioritized systemic, logistical, social, and inter-/intrapersonal obstacles are essential for improving access and optimizing mental health care outcomes. These insights underscore the complexity of considerations for Veterans and families accessing mental health care.

## Introduction

For some Veterans, the post-service period is characterized by complex challenges (1, 2). Indeed, over one-third of Canadian Armed Forces (CAF) Veterans report a moderate or very difficult transition to civilian life (3). The reintegration experience involves significant changes to one's physical environment (e.g., work, housing) and identity (e.g., role within the family, relationships), which impose new duties, expectations, and stressors. A difficult transition to civilian life has been associated with poor mental health outcomes (2), including an increased risk of mental disorders and suicide (4, 5). Importantly, these changes are embedded within larger socio-ecological contexts and are amplified by existing health disparities and inequities.

While the perceived need for mental health care increases following the transition from the military to civilian life, St. Cyr et al. (6) found that a similar percentage of active members of the CAF and Canadian Veterans report accessing mental health care in the previous year. However, Veterans may experience different barriers to mental health care than actively serving military personnel, including geographical barriers to care (vs. having health care services readily available on base) and a lack of resources or knowledge of available support for Veterans [e.g., (7–9)]. Further, certain aspects of military culture, such as a strong emphasis on self-reliance, may be integrated into Veterans' core beliefs (10, 11) and deter help seeking. Additionally, the interpersonal, psychological, and behavioural difficulties that may be experienced during the post-service transition may also serve as barriers to treatment-seeking in this population (9, 12). Finally, it is important to note that following the transition to civilian life, access to certain services and supports (e.g., unit support, military-specific mental health care) may become more limited or stop (13, 14). Importantly, research has found a positive relation between mental health care access and health status among Veteran population [see (15)], which may also extend to the families of transitioning Veterans given the experiential link between Veterans and their family members [e.g., (9)]. Indeed, Schwartz et al. (9) note that many of the aforementioned barriers also impact Veteran family members, although the severity of their impact may vary with respect to various individual and environmental factors (9). Further, Maguire et al. (16) found that many health and wellbeing needs of Veteran families are amplified during the transition from military service to civilian life, and these families often face challenges navigating civilian systems of care. Indeed, Veterans and their families may encounter additional difficulties relative to active duty military personnel, especially related to continuity of care following release from the military when sources of health care and benefits change as a result of Veteran status [e.g., from the Department of Defense to Veterans Affairs; (17)]. It is therefore critical to elucidate existing barriers and facilitators to mental health care among Veterans and Veteran families to understand gaps in service access or experiences [including across demographic characteristics, such as gender; see Cornish et al., (12)].

To inform health care planning and policies within Canada, it is necessary to explore contemporary literature which describes experiences among similar populations of Veterans and families. As such, examining literature from across the Five Eyes nations (i.e., Australia, Canada, New Zealand, United Kingdom, United States), which are all Westernized countries sharing important similarities such as governmental structure and historical and military alliances [as noted in (18)], broadens the scope of available information when considering experiences of miliary personnel and families. Further, a representative sample across these countries allows for examinations of certain between-country nuances (e.g., health care delivery systems) in a review context [e.g., (19)]. For example, one report [see (20)] noted high discrepancies in budgets for expenditures, and the number of case managers and staff available to Veterans across various allied nations, among other findings.

Given the complexity of barriers and facilitators affecting mental health service access for Veterans and their families, there is a need for a structured, theory-based approach to identify and address these critical factors effectively. The Theoretical Domains Framework [TDF; (21, 22)] offers a particularly effective method for this purpose. The TDF an integrative framework used to support implementation objectives by providing a sound theoretical basis for assessing behavioural influences and promoting behavioural change to improve outcomes in various clinical contexts. The TDF outlines 14 domains (e.g., knowledge, skills, optimism, beliefs about consequences, goals, etc.) which include 84 component constructs that further specify aspects of each domain [e.g., within the domain of knowledge: procedural knowledge, knowledge of task environment, and other relevant knowledge; (22)]. These domains, in turn, influence physical, psychological, social, automatic, and reflective sources of behaviours that contribute to one's capability, opportunity, and motivation, which interact to produce behaviour (21). Notably, the TDF can be applied both deductively (e.g., as a preliminary framework for content analysis) and inductively [e.g., to generate themes relative to domains; (21)]. In the present review, the inductive utility of TDF was applied to identify barriers and facilitators which might influence treatment-seeking behaviours among Veterans and their families. The TDF has been successfully used to assess barriers and facilitators across different areas of health care [e.g., (23, 24)]; this review extends its use to specifically address the empirical literature concerning mental health service access by Veterans and families, providing a novel insight into this context.

Existing systematic reviews examining health-related behaviours among Veterans either focus exclusively on quantitative research (25) or were specific to help-seeking behaviours (26) which are distinct from actual access to care. Further, existing reviews do not contextualize findings within a validated framework. Taken together, the aim of this review is to identify barriers and facilitators to mental health care by examining the lived experiences (via qualitative and quantitative data) of Veterans and their families accessing mental health care during the post-service period, through the lens of the TDF. Findings from this review can be used to highlight considerations or inform actionable recommendations related to health policy for Canadian Veterans. Specifically, this review aimed to address the following research questions: (1) How do Veterans and family members articulate barriers and facilitators to access to and reception of mental health services during the post-service period?; (2) What factors may optimize access to and reception of mental health services for Veterans and families during the post-service period?; (3) What considerations may need to be in place at the policy level to facilitate changes to better promote mental health access and care for Veterans and their families during the post-service period?; and (4) How does mental health interact with other domains of wellbeing (as represented in the TDF)?

## Methods

This review was conducted using the Cochrane Handbook for Systematic Reviews of Interventions as a methodological framework (27). Cochrane guidelines were chosen as they are internationally regarded for their transparency, standardized and replicable methodologies, and methodological rigour across a variety of health and health related disciplines synthesizing both quantitative and qualitative data [e.g., (28–30)]. Indeed, one survey of Moseley et al. (31) found that reviews implementing Cochrane Collaboration procedures demonstrated higher rigour and overall quality. The review process included deploying a search strategy across multiple databases, two levels of screening (title and abstract and full text) against inclusion and exclusion criteria, resolving conflicts at each level, as well as data extraction, data analyses, and data synthesis. This review followed the Preferred Reporting Items for Systematic Review and Meta-Analysis - Rapid Review (PRISMA-RR) guidelines for standards of reporting findings (32).

### Search strategy

We conducted a preliminary search for ten relevant articles that should be included in a systematic literature search. These articles served as “benchmark articles” to ensure our search strategy was accurate and comprehensive across a number of indexing databases (33). Our team then consulted with an academic librarian at Western University to confirm the following search strategy. In light of optimizing databases across the benchmarking articles and meeting the minimum number of searched databases required for systematic reviews [e.g., (34, 35)], we selected three databases to perform our search: Scopus, Medline (OVID), and PsycINFO (ProQuest). The following keywords were identified: military, “armed forces”, soldier, RCMP, Veteran*, transiti*, retir*, resources, “mental health care”, programs, “mental health service use”, “mental health services use”, “mental health care”, “mental health support”, “mental health treatment”, “mental health use”, “psychiatr* service use”, “utili*ation”, “help-seeking”, and “mental health” (see Supplementary Material for string terms). We imposed a date restriction of 10 years from the search date (i.e., 2013–2023), which was conducted on December 18, 2023, in order to focus on contemporary barriers and facilitators of mental health care. In addition, the Veterans Affairs Canada (VAC) Research Directorate webpage was scanned to identify any potentially relevant grey literature.

### Inclusion and exclusion criteria

Articles were included if they:

(1) focused on military Veterans who have been released from service for any reason,
(2) focused on families of Veterans,
(3) reported on post-service experiences and mental health service use, and
(4) described barriers and/or facilitators to accessing and/or using mental health services.

Articles were excluded if they:

(1) did not differentiate between active duty and Veteran populations,
(2) reported the occurrence of a barrier or facilitator without any context of any specific barrier or facilitator,
(3) exclusively focused on medical services, including chronic pain,
(4) were non-study papers (e.g., books, news articles),
(5) not available in English or French,
(6) were not from the Five Eyes Countries [Canada, United States, United Kingdom (UK), Australia, New Zealand], and
(7) were published before 2013.

With respect to exclusion criterion (7), the goal of this review was to be able to provide actionable policy recommendations related to mental health service use by Veterans and their families. As such, this review will focus on capturing relatively contemporary barriers and facilitators to mental health care. Further, given that rapid reviews are often conducted to support policy-focused work [e.g., (36)], a 10-year search restriction allowed for the requisite timely execution of this style of review in consideration of the scope of available literature.

### Study selection

**Figure 1:**
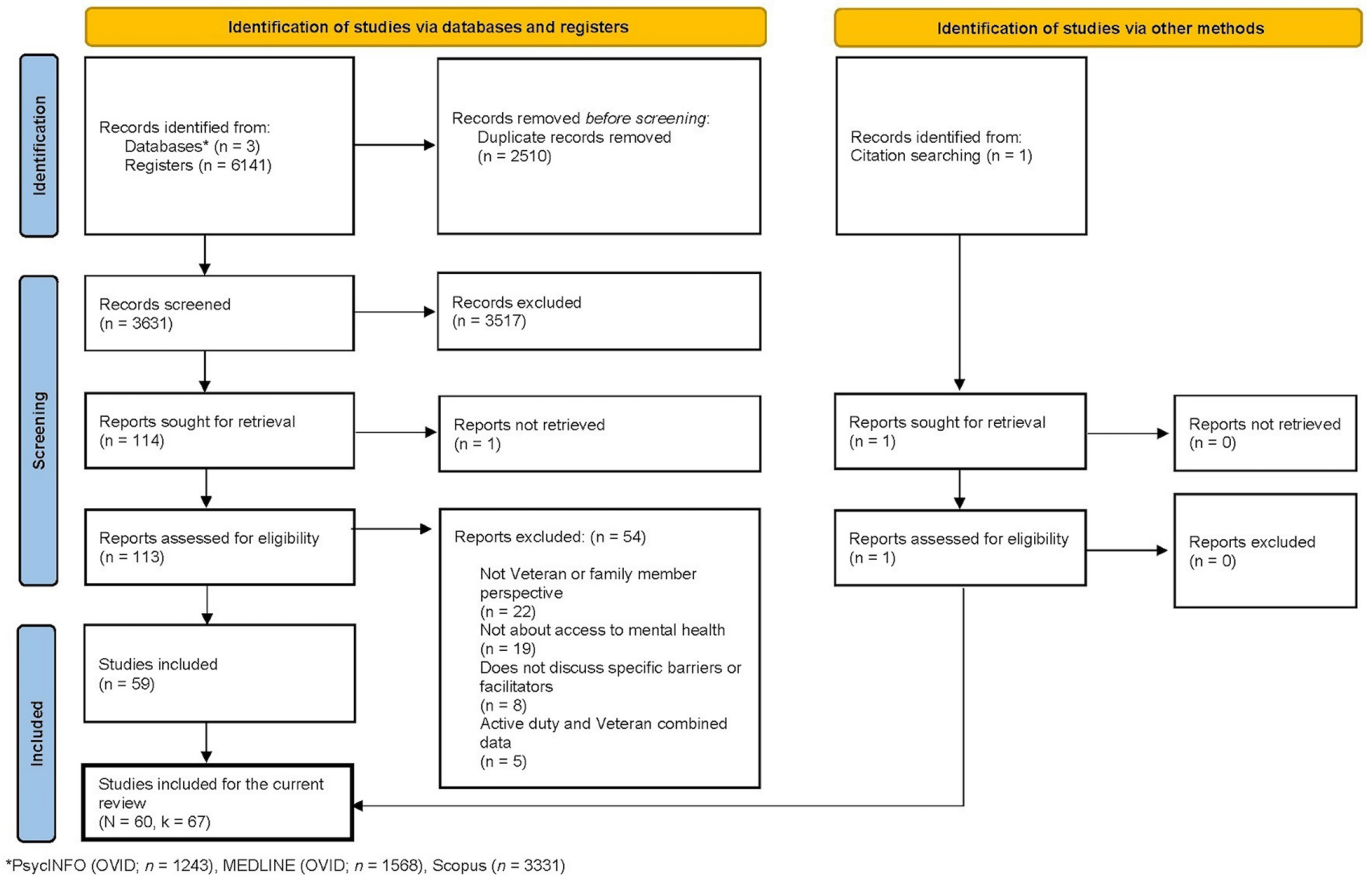
Preferred reporting items for systematic reviews and meta-analysis.

Following the deduplication of database outputs, two screeners independently reviewed each article against the inclusion and exclusion criteria for title and abstract review and full-text review. Across the entire body of citations, seven screeners participated in the screening process. Interrater reliability was good for title and abstract review [using percent agreement (97.2%), and Kappa (Fleiss and Conger; 0.742)] and full-text review [using percent agreement (90.4%), and Kappa (Fleiss and Conger; 0.705)]. At the data extraction stage, two raters extracted relevant information from included articles. Conflicts were resolved at each level of screening by study authors until a consensus was reached (see Figure 1). SWIFT-Active Screener, a web-based, collaborative review software that accelerates the screening processes, was used for the title and abstract and full-text review stages. SWIFT enlists a proprietary machine-learning algorithm to prioritize relevant articles for screening with a high degree of accuracy (37). Using this approach, our review and screening times were reduced without risk or loss of accuracy.

### Data extraction

The following information was extracted from each article (1): article and demographic information, and (2) barriers and facilitators to mental health service use reported by Veterans or their family members. For article and demographic information, the following data were extracted: *type of article* (i.e., empirical article or dissertation/theses, further broken down by qualitative, quantitative or mixed data), *country of study* (i.e., Canada, United Kingdom, United States, New Zealand, Australia), *age, gender/sex* (i.e., mixed, women/female, men/male, transgender, not specified), *race* (i.e., White/European, Black/African/Caribbean, East Asian, Southeast Asian, Hawaiian/Pacific Islander, Indigenous Peoples, Latin American/Hispanic, Multiracial/Multiethnic, other, mixed, not specified), *income* (i.e., less than $40,000, $40,000–$59,999, $60,000–$79,999, $80,000–$99,999, $100,000–$119,999, $120,000 or more, mixed, not specified), *education* (i.e., less than high school, high school, college, undergraduate, graduate/professional degree, mixed, not specified), *length of service, type of release* (i.e., honourably discharged, dishonourably discharged, not specified), number of *years since release,* and *Veteran* vs. *family perspective* (i.e., Veteran, family, both).

Barrier and facilitator information associated with mental health service use (as reported by Veteran or their family members) was extracted from each article. To be considered a barrier, the results of the study must have explicitly stated an obstacle, difficulty, or challenge Veterans and/or their family members experienced during access to mental health service use. To be considered a facilitator, the results of the study must have explicitly stated a factor that eased the access to mental health service use for Veterans and/or their family members. As such, two independent reviewers extracted the data in the following stages:

○ *Step 1.* Extracted direct quotes or empirical outcome data from the article and categorized as being experienced by a Veteran or family member.
○ *Step 2.* Examined the raw data and identified common, repeated themes from a policy informed lens and categorized into barriers or facilitators within the dataset.
○ *Step 3.* Systematically grouped the emerging themes and subsequently organized them into relevant categories (e.g., Barriers–Veterans; Barriers–family members; Facilitators–Veterans; Facilitators–family members; see Table 1 for descriptive information about the emerging themes). For the primary analysis, a thematically-driven approach was used to classify barriers and facilitators to mental health care, rather than a pre-existing theoretical framework, to ensure a good fit of the data. For the secondary analysis, we mapped barriers and facilitators onto the domains of the TDF.

**Table 1:** Definition of emerging themes across barriers and facilitators.

Themes	Socioecological domain	Description	Examples
Barriers			
Gaps in mental health knowledge	Inter-/Intrapersonal	Waiting until symptoms are severe before seeking treatment, not understanding the early signs of mental health issues	“Some veterans did not consider that they had the knowledge or understanding to confidently label their distress as being mental health related. Others stated that the lack of perceived severity, or their ability to self-manage symptoms, meant that they were not a mental disorder.” (38)
Inadequate sense of safety	Inter-/Intrapersonal	Not feeling safe in a mental health care environment- experiences such as harassment from peers, discrimination	“I’m just so tired. I hate, I hate going to the VA. The doctors are great, but I hate going up there. I hate it. It's nothing but men. I was verbally assaulted with one of the guys that works in the cafeteria.” (39)
Lack of trust in provider	Inter-/Intrapersonal	Uncomfortable discussing personal experiences with provider, does not feel that provider has their best interest in mind	“If you start talking about certain things that you can't even talk to your wife about without getting emotional, I really didn't feel comfortable doing that with someone I didn't even know … so, that was kind of a big thing that kept me from talking to somebody about it for a long time.” (12)
Lack of trust in the system	Inter-/Intrapersonal	Fear of a breach in confidentiality, does not believe in the mental health care system's mission or ability to ease mental health conditions	“I got out of the service in 1982, something like that, but I’d never been to the VA because I was always under the impression that nobody cared. Nobody really wanted to help people.” (11)
Negative pre-existing attitudes & beliefs	Inter-/Intrapersonal	Stigma and negative experiences with mental health services	“One participant said, ‘[If I sought help] he's going to think I’m weird. He's going think I’m different … You don't want people to view you differently. Nobody wants to be an outcast.” (12)
Unwanted emphasis on military identity throughout services	Inter-/Intrapersonal	Not wanting to be recognized as a Veteran upon retirement and/or not proud of their military experience (do not want recognition)	“This is not targeted toward me […] I’m going to retire and grow my hair out and be quiet about this”. (Sierra). For some veterans, “the identity of being a vet can feel oppressive at times.” (40)
Costly services & travel	Logistical	Cost of services and travel	For some participants, the cost of services was identified as a barrier to seeking mental health care. For example, Participant Seven said; “Well if it was free, that would be tremendous benefit.” (41)
Lifestyle disruptions	Logistical	Constraints such as getting time off work, not having access to childcare, caregiving responsibilities	“OEF/OIF veterans were more likely than Vietnam veterans to agree that work conflicts interfere with treatment and their lives are too busy for treatment.” (42)
Transportation challenges	Logistical	Unable to access a vehicle or transit system to get to a mental health service location and/or mental health service location is far away	“Some veterans mentioned that the local van (that provides transportation to VA services) was too limited to provide transportation to the services they needed.” (43)
Fear of repercussions	Social environment	Fear of negative consequences (e.g., being discharged)	“…might defer seeking treatment for fear of jeopardizing their chances for career advancement.” (44)
Gender stereotype	Social environment	–	“I know a lot of female veterans that are struggling with mental health and they’re just being pushed to the side and the men are being taken care of and the women are just being shoved and forgotten. P1 felt dismissed by her GP as just a “depressed woman, has children, probably struggling that way” rather than a veteran with potentially complex needs.” (45)
Lack of social support	Social environment	Not having emotional support from family or friends	“Once you get out, as we talked about throughout, you don't have that support system anymore.” (46)
Military culture of stoicism	Social environment	A culture of stoicism and self-reliance creating a “tough it out” attitude towards mental health	“Veterans also spoke of not deserving care, stemming from the military ethos of being self-reliant and not burdening anyone else with their individual problems.” (38)
Unwanted social/Organizational pressure	Social environment	Feeling pressure from family/friends or organizations to seek mental health care	“It took a long time for Veteran One to come back to the VA; she felt as though the VA dangled her disability rating over her head to make her come back and receive treatments.” (47)
Difficulty navigating the system	Systemic	Difficulty completing paperwork/forms and understanding benefit services, technological difficulties, poor knowledge of available services	“I spent six months filling out the same form four times and getting it sent back to me saying it was filled out wrong. So, i learned to cope on my own because I wasn't getting help [with the VA].” (40)
Lack of continuity of care	Systemic	Difficult transition from Canadian Armed Forces (CAF) to Veteran Affairs Canada (VAC), different providers, provider turnover	“Veterans dislike high clinic staff turnover. Veterans were disappointed with high provider turnover, resulting in many “first appointments” and in some cases the disruption of long-standing therapeutic relationships.” (48)
Lack of provider engagement	Systemic	Noticeable disinterest or minimal involvement from provider in actively participating in or contributing to care and therapeutic processes	“Many described appointments where a provider appeared disinterested, avoided eye contact, or seemed impatient.” (49)
Lack of service availability	Systemic	Service was not accessible/available	“Very often the individual has to tough it out because there is no help available.” (50)
Lack of service preference	Systemic	Lacking patient choice of care and provider and lack of gender-specific care	“The care I wanted was too difficult to get in VA I did not think I would be able to participate in women-only group treatments as often as I liked in VA.” (51)
Long wait times	Systemic	–	“Six month to a two year waiting list.” (41)
Provider unfamiliar with military culture & patient condition	Systemic	Provider lacks knowledge about military life and experiences and/or lacks expertise on the patient's condition	“These professionals that are supposed to know about mental health they haven't got a clue about soldiers’ mental health because what affects a civilian for their mental health issues is not the same for a military person.” (45)
Symptoms limiting care accessibility	Systemic	Unable to access care due to severity of mental health and/or co-occurring symptoms e.g., exhaustion, pain	“Veterans also described how co-occurring health conditions, such as cancer, drowsiness, depression, and headaches, interfered in their ability to attend treatment.” (52)
Unwanted emphasis on pharmaceutical treatment by provider	Systemic	Provider does not take into account patient preferences for nonpharmaceutical treatment when providing mental health services	“I don't do well with medication. I’ve already had anger issues and (when taking drugs) I become ballistic! (laughter). I tell them, I’m not the right person, you know. But they’re like, “oh well, try this one”, or they say “you need at least six months on this”. So I just stopped.” (49)
Facilitators			
Mental health knowledge	Inter-/Intrapersonal	Understanding of mental health, mental health symptoms and/or the therapeutic process	“She said that “[the doctor] was able to persuade me that it wasn't weakness to go and see someone” and that health care professionals “tried to re-educate me” in realizing that a military mind-set on ill health was no longer appropriate and was potentially acting as a barrier to help-seeking.” (45)
Trust in provider	Inter-/Intrapersonal	Ability to confide in provider without fear of judgement, believes that provider has their best interest in mind	“They’re helping me and they’re concerned … [If I were having a bad day] I’d probably try to call [provider] and try to get ahold of her because she's told me, you know, call me if you’re having problems so I know I can do that.” (11)
Trust in the system	Inter-/Intrapersonal	Belief in the effectiveness of mental health treatment system, confidentiality	“Previous treatment experiences played a role in mitigating negative perceptions around the utility of treatment for some veterans. It was a lot easier to reach out because I already knew that reaching out would help.” (38)
Affordable cost	Logistical	Good or sufficient benefits, low- or no- fee services	“Participant 5 described how free counseling services offered through her local Military Family Resource Centre eliminated financial and insurance-related barriers, thereby facilitating continued engagement: I was able to go on my time. I would just continue going, it never ran out, I never had benefits that ran out.” (53)
Childcare/transportation availability	Logistical	Childcare, transportation services	“She stated shuttle buses would meet at different locations to drive veterans to the hospital for their appointments, which was helpful.” (47)
Convenience	Logistical	Close proximity of mental health services, mental health services in the same facility as physical health services	“ONCE I found [the local VA clinic] I was like oh! Ok! I can come here for both my physical and my mental? Because when I first started coming here, it was for my pain to get a new doctor. And then I started with the mental health services and I went “Sweet!” It was very, very helpful to have them both collocated.” (54)
Social support	Social environment	Emotional support from family and peers	“For some partners, the veteran's supportiveness of continued use not only served to reaffirm the value and benefits of service engagement but also generated a sense of mutual accountability to managing the impact of PTSD together.” (53)
Continuity of care	Systemic	Smooth transitions between providers and levels of care, follow up	“They gave me a number to ring up all the time, I kept getting the weekly visits from the regional welfare officer, and probably every couple of months from the community practice nurse … Nothing was too much trouble for them.” (55)
Low wait times	Systemic	Timely access to care	“So I got in touch with the service, they were brilliant dead quick really really quick.” (56)
Positive service environment	Systemic	positive service environment (e.g., professionalism, cleanliness)	“Partners also expressed the importance of a welcoming, warm service atmosphere.”
Provider engagement	Systemic	Provider is attentive and actively listens	“She was very comfortable and everything, and really open. She would always, uh, she had a good, like, engagement style, like, her body language was very open. […] she was always seeing the true intent of what I was saying and obviously listening.” (57)
Provider familiar with military culture & patient condition	Systemic	Provider has knowledge of military culture, experiences, and/or expertise on the client's condition	“Participants described it being easier to communicate with therapists who understood military culture. ‘When I saw her it was really really good because yes she had a little bit of understanding.” (56)
Service availability	Systemic	Availability of services (e.g., types of treatment available, flexible timing such as weekend hours), ability to access non-military affiliated care and alternative treatments (e.g., mental health retreats), telehealth, choice of provider, engagement of family members in care	“For some partners, consistent flexibility with work hours and schedules reassured them that they would be able to balance the demands of their work and home life with the time commitment needed to reach their desired therapeutic goals.” (53)
Support navigating the system	Systemic	Support with paperwork/forms, finding a provider	“Women veterans also shared positive experiences, such as when they received help from older veterans or officials at the tribe who had a better grasp of the paperwork process as well as learning how to maneuver within the system.” (43)

### Risk of bias assessment

The Mixed Method Appraisal Tool [MMAT; (58)] was used to evaluate the risk of bias among articles. The MMAT is a valid and efficient quality assessment tool that allows for simultaneous appraisal of qualitative, quantitative, and mixed methods studies. Each article was categorized by study type (i.e., qualitative, quantitative, or mixed methods). Studies were then rated based on the MMAT criteria corresponding to the respective study type. Criteria included five specific appraisal questions assessing methodological characteristics (e.g., appropriate and effectively executed methodological approach) relative to the type of study conducted. Criteria were evaluated with “yes”, “no”, and “can’t tell” answer options.

### Data analysis

Smartsheet and Microsoft Excel were used for data analyses. Demographic information was examined using descriptive statistics (i.e., frequencies; means and standard deviations). The themes emerged through deductive analysis based on our own binning process. Initially, themes were identified and categorized as either barriers or facilitators. These themes were then thematically organized into socio-ecological domains. This organization was based on the structural, environmental, or individual level at which these barriers and facilitators were described as relevant: (1) *Systemic* which refers to the obstacles or benefits that are embedded within the structure, policies, or practices of a system that impacts one's access to mental health services, (2) *Inter-/Intrapersonal* which refers to obstacles or benefits arising from interactions between individuals or within an individual's own thoughts, beliefs, or behaviors which influence help-seeking for mental health care, (3) *Logistical* which refers to obstacles or benefits that arise from practical considerations such as transportation, scheduling, or infrastructure limitations related to accessing mental health services, and (4) *Social Environment* which refers to obstacles or benefits originating from an individual's physical surroundings, social interactions, or external influences, which influence help-seeking for mental health care. While an emerging theme may recur throughout an article, our results are focused on the identification of unique themes within each article, rather than the frequency with which themes are repeated within an article.

Barriers and facilitators were then thematically organized across the 14 domains of the TDF: (1) *Knowledge w*hich refers to being aware of the existence of something, (2) *Skills* which refer to abilities or proficiencies acquired through practice, (3) S*ocial/Professional Role and Identity* which refers to the behaviours and qualities that individuals display in social or work settings, (4) *Beliefs about Capabilities* which refers to the level of acceptance of an ability that a person can put to use, (5) *Optimism* which refers to the level of belief that a goal can be attained or that something will happen for the best, (6) *Beliefs about Consequences* which refers to the level of acceptance for the outcomes of a behaviour in a particular setting, (7) *Reinforcement* which refers to the arrangement of a dependent relationship or contingency between a stimulus and response which increases the odds of that given response, (8) *Intentions* which refers to the decision that one makes to perform a behaviour in a certain manner, (9) *Goals* which refers to a mental representation of an end-state that an individual seeks to achieve, (10) *Memory, Attention, and Decision Processes* which refers to the ability to focus selectively, retain information, or make a choice between alternatives, (11) *Environmental Context and Resource* which refers to circumstances of an individual's environment or situation that either promote or discourage the development of abilities, independence, or adaptive behaviours, (12) *Social Influences* which refers to interpersonal processes that can lead individuals to change their behaviours, thoughts, or feelings, (13) *Emotion* which refers to reactionary patterns involving experiential, behavioural, and physiological elements to deal with a significant matter or event, and (14) *Behavioural Regulation* which refers to anything that is aimed at changing or managing measured or observed actions.

MMAT scoring to assess the risk of bias was calculated based on the percentage of MMAT criteria met (i.e., number of “yes” responses). Hong et al. (58) noted that calculating an overall score is not advisable. As such, studies were rated based on the percentage of criteria met [i.e., 20, 40, 60, 80, 100; per (58)].

## Results

### Study characteristics

The final sample consisted of 60 articles (denoted with *N*) with 67 independent samples (denoted with *k*; Figure 1; see Supplementary Material for raw data and reference list of included articles). The samples mostly included Veteran (*k *= 58) perspectives, with a few from family perspectives (*k *= 9). Across all included articles, this review identified 23 barriers and 14 facilitator themes. It is important to note that the demographic characteristics reported in Table 2 do not fully represent the participants across samples due to inconsistencies in the information reported across the included samples (see Table 2 for characteristic information). Notably, across all samples, 11 articles discussed topics related to military sexual trauma.

**Table 2:** Study characteristics (*N* = 60).

Characteristic	*N*	%
Type of article	–	–
Empirical article	44	73
Dissertation/theses	16	27
Type of data	–	–
Qualitative	41	68
Quantitative	15	25
Mixed	4	7
Country of study	–	–
United States	49	82
United Kingdom	7	12
Canada	4	6
Australia	0	0
New Zealand	0	0
Age	–	–
Not specified	46	77
Specified (mean age)	14	23
Gender/Sex	–	–
Mixed	32	53
Women/Female	16	27
Men/Male	10	17
Not specified	2	3
Race	–	–
Mixed	45	75
Not specified	15	25
Income	–	–
Not specified	52	87
Mixed	8	13
Education	–	–
Not specified	43	72
Mixed	17	28
Length of service	–	–
Not specified	42	70
Specified (mean age/range)	18	30
Type of release	–	–
Not specified	57	95
Honourably discharged	3	5
Year since release	–	–
Not specified	55	92
Specified (mean age/range)	5	8

### Examination of barrier and facilitator themes across socio-ecological domains

#### Veteran perspective

Across all samples that captured the Veteran's perspective (*k* = 58), the most common type of barrier experienced was systemic in nature (*n *= 146; in this section, *n* denotes the total number of barriers or facilitators reported across all included articles within a respective domain as per Table 3). Within this domain, the most commonly identified theme was difficulty navigating the system (e.g., difficulty completing forms; *k *= 38; 66%), followed by health care provider (i.e., physician, clinical psychologist, or psychotherapist) unfamiliar with military culture and patient condition (e.g., provider lacks knowledge about military life; *k *= 25; 43%), and lack of service preference (e.g., lacking patient choice of care; *k *= 24; 41%). The second most common domain was inter-/intrapersonal (*n *= 112). The most common theme identified within this domain was negative preexisting attitudes and beliefs towards mental health (e.g., stigma; *k *= 35; 60%), followed by gaps in mental health knowledge (e.g., not understanding the early signs of mental health issues; *k *= 32; 55%), and lack of trust in the system (e.g., belief that the mental health care system cannot ease mental health conditions; *k *= 21; 36%). The third most common domain was logistical (*n *= 47). In this domain, the identified themes exhibited relatively equal prevalence: transportation challenges (e.g., location of mental health service is too far away; *k *= 16; 28%), lifestyle disruptions (e.g., not able to take time off from work; *k *= 16; 28%) and costly services and travel (e.g., cost of service is too expensive; *k *= 15; 26%). Social environment was the least identified domain (*n *= 46). Within this domain, the most common theme was a military culture of stoicism (e.g., a culture creating a “tough it out” attitude; *k *= 22; 38%), followed by fear of repercussions (e.g., fear of negative consequences such as losing out of job opportunities; *k *= 12; 21%), and gender stereotypes (*k *= 12; 21%).

**Table 3:** Characteristics of barriers and facilitators across Veterans and their families (*k* = 67).

Themes	*k* (%)
Veteran (*k* = 58)	
Barriers	
Systemic (*n* = 146)	
Difficulty navigating the system	38 (66)
Provider unfamiliar with military culture & patient condition	25 (43)
Lack of service preference	24 (41)
Long wait times	15 (26)
Lack of continuity of care	14 (24)
Unwanted emphasis on pharmaceutical treatment by provider	9 (16)
Lack of provider engagement	8 (14)
Symptoms limiting care accessibility	8 (14)
Lack of service availability	5 (9)
Inter-/Intrapersonal (*n *= 112)	
Negative pre-existing attitudes & beliefs	35 (60)
Gaps in mental health knowledge	32 (55)
Lack of trust in the system	21 (36)
Lack of trust in provider	17 (29)
Inadequate sense of safety	6 (10)
Unwanted emphasis on military identity throughout services	1 (2)
Logistical (*n *= 47)	
Transportation challenges	16 (28)
Lifestyle disruptions	16 (28)
Costly services & travel	15 (26)
Social environment (*n *= 46)	
Military culture of stoicism	22 (38)
Fear of repercussions	12 (21)
Gender stereotype	12 (21)
Lack of social support	5 (9)
Unwanted social/organizational pressure	3 (5)
Facilitators	
Systemic (*n *= 58)	
Support navigating the system	16 (28)
Service availability	14 (24)
Provider familiar with military culture & patient condition	12 (21)
Continuity of care	7 (12)
Provider engagement	5 (9)
Positive service environment	3 (5)
Low wait times	1 (2)
Inter-/Intrapersonal (*n *= 21)	
Mental health knowledge	12 (21)
Trust in the system	5 (9)
Trust in provider	4 (7)
Social environment (*n *= 17)	
Social support	17 (29)
Logistical (*n *= 6)	
Convenience	3 (5)
Childcare/transportation availability	2 (4)
Affordable cost	1 (2)
Family (*k* = 9)	
Barriers	
Systemic (*n *= 12)	
Difficulty navigating the system	5 (56)
Lack of service preference	3 (33)
Provider unfamiliar with military culture & patient condition	2 (22)
Lack of continuity of care	1 (11)
Lack of service availability	1 (11)
Long wait times	0 (0)
Lack of provider engagement	0 (0)
Symptoms limiting care accessibility	0 (0)
Unwanted emphasis on pharmaceutical treatment by provider	0 (0)
Inter-/Intrapersonal (*n *= 11)	
Gaps in mental health knowledge	4 (44)
Negative pre-existing attitudes & beliefs	3 (33)
Lack of trust in provider	2 (22)
Lack of trust in the system	2 (22)
Unwanted emphasis on military identity throughout services	0 (0)
Inadequate sense of safety	0 (0)
Logistical (*n *= 8)	
Lifestyle disruptions	4 (44)
Transportation challenges	2 (22)
Costly services & travel	2 (22)
Social environment (*n *= 1)	
Lack of social support	1 (11)
Gender stereotype	0 (0)
Military culture of stoicism	0 (0)
Fear of repercussions	0 (0)
Unwanted social/Organizational pressure	0 (0)
Facilitators	
Inter-/Intrapersonal (*n *= 6)	
Mental health knowledge	4 (44)
Trust in the system	1 (11)
Trust in provider	1 (11)
Systemic (*n *= 5)	
Service availability	2 (22)
Positive service environment	1 (11)
Support navigating the system	1 (11)
Provider familiar with military culture & patient condition	1 (11)
Provider engagement	0 (0)
Continuity of care	0 (0)
Low wait times	0 (0)
Social environment (*n *= 4)	
Social support	4 (44)
Logistical (*n *= 2)	
Childcare/Transportation availability	1 (11)
Affordable cost	1 (11)
Convenience	0 (0)

*n* reflects the total number of barriers or facilitators reported across all included articles within each domain.

As for facilitators reported by Veterans, similarly, the most common domain was systemic (*n *= 58). Specifically, support navigating the system was the most prominent theme (*k *= 16; 28%), followed by service availability (*k *= 14; 24%), and health care provider familiarity with military culture and patient condition (*k *= 12; 21%). The second common domain was inter-/intrapersonal (*n *= 21). In particular, the most common themes were mental health knowledge (*k *= 12; 21%), followed by trust in the system (*k *= 5; 9%), and trust in provider (e.g., feeling the provider has their best interest in mind; *k *= 4; 7%). The third most common domain was social environment, with social support being the only emerging theme (*k *= 17; 29%). Lastly, logistical was the least prevalent domain (*n *= 6). Within this domain, the reported themes included convenience (e.g., mental health service is close; *k *= 3; 5%), childcare/transportation availability (*k *= 2; 4%), and affordable cost (*k *= 1; 2%; see Table 3 for reported themes within each domain across barriers and facilitators, as experienced by Veterans).

#### Family perspective

Similarly, across all samples that captured the Veteran family's perspective (*k* = 9), the most common barrier was systemic (*n *= 12). Specifically, difficulty navigating the system was reported as the most prominent theme (*k *= 5; 56%), followed by lack of service preference (*k *= 3; 33%) and provider unfamiliar with military culture and patient condition (*k *= 2; 22%). The inter-/intrapersonal domain was reported with almost equal frequency (*n *= 11). Within this domain, gaps in mental health knowledge was the most prominent barrier (*k *= 4; 44%), while negative preexisting attitudes and beliefs (*k *= 3; 33%), trust in provider (*k *= 2; 22%), and trust in the system (*k *= 2; 22%) were equally common. The third most common domain was logistical (*n *= 8). In particular, lifestyle disruptions were most common (*k *= 4; 44%), followed by transportation challenges (*k *= 2; 22%) and costly service and travel (*k *= 2; 22%) reported as equally common. The final domain, social environment, was not a prominent domain within the family literature (*n *= 1).

With regards to facilitators, the most prominent domain was inter-/intrapersonal (*n *= 6). Within this domain, the only repeated theme was mental health knowledge (*k *= 4; 44%). The next most common domain was systemic (*n *= 5), with service availability (*k *= 2; 22%) being the only repeatedly reported theme. Importantly, the social environment domain found social support was the only theme reported (*k *= 4; 44%). Lastly, the logistical domain was not prominent (*n *= 2; see Table 3 for reported themes within each domain across barriers and facilitators, as experienced by families).

### Examination of the barriers and facilitators within the Theoretical Domains Framework

When examining the themes within the TDF framework, six of the domains were captured by the data in this review: knowledge, social/professional role and identity, beliefs about capabilities, beliefs about consequences, environmental context and resources, and social influences. Most of the 23 identified barriers were categorized into environmental context and resources (*n *= 10; 43%; in this section, *n* denotes the number of barrier or facilitator themes across TDF domains per Table 4), followed by social influences (*n *= 4; 17%), knowledge (*n *= 3; 13%) and beliefs about consequences (*n *= 3; 13%), social/professional role and identity (*n *= 2; 9%), and beliefs about capabilities (*n *= 1; 4%). As for facilitators, most of the 14 identified facilitators were embedded into the environmental context and resources (*n *= 7; 50%), followed by knowledge (*n *= 3; 21%), beliefs about consequences (*n *= 2; 14%), social/professional role and identity (*n *= 1; 7%) and social influences (*n *= 1; 7%).

**Table 4:** Barrier and facilitators themes embedded into Theoretical Domains Framework.

Framework	Barriers	Facilitators
Knowledge	Difficulty navigating the system Gaps in mental health knowledge Provider unfamiliar with military culture & patient condition	Support navigating the system Mental health knowledge Provider familiar with military culture & patient condition
Skills	–	–
Social/Professional role & identity	Unwanted emphasis on military identity throughout services Lack of provider engagement	Provider engagement
Beliefs about capabilities	Symptoms limiting care accessibility	–
Optimism	–	–
Beliefs about consequences	Fear of repercussions Lack of trust in the system Lack of trust in provider	Trust in the system Trust in provider
Reinforcement	–	–
Intentions	–	–
Goals	–	–
Memory, attention & decision processes	–	–
Environmental context & resources	Long wait times Lack of continuity of care Lifestyle disruptions Transportation challenges Lack of service availability Lack of service preference Costly services & travel Inadequate sense of safety Unwanted emphasis on pharmaceutical treatment by provider Unwanted social/Organizational pressure	Positive service environment Low wait times Continuity of care Convenience Childcare/Transportation availability Affordable cost Service availability
Social influences	Military culture of stoicism Lack of social support Gender stereotype Negative pre-existing attitudes & beliefs	Social support
Emotion	–	–
Behavioural regulation	–	–

### Risk of bias (Mixed Method Appraisal Tool)

Overall, the methodological quality of the included articles (*N *= 60) varied: 52% of the articles met ≤60% of MMAT criteria (31/60; 20% [*n *= 3], 40% [*n *= 12], 60% [*n *= 16]), while 48% of the articles met >60% of MMAT criteria (29/60; 80% [*n *= 28], 100% [*n *= 1]). Notably, the most common criterion was a quality threshold of 80% (see Supplementary Material for MMAT scores of all included articles).

## Discussion

### Emerging barrier and facilitator themes within the socio-ecological domains

Veterans and Veteran family members identified a number of common barriers and facilitators across systemic, interpersonal/intrapersonal, logistical, and social environmental domains.

#### Systemic

Systemic barriers were the most cited barriers to accessing mental health care with issues navigating the system reported by over half of the Veteran samples. Upon retirement, Veterans move from a military-specific health care system to the provincial or territorial health care system in their province or territory of residence, with additional VAC health care benefits being available for Veterans who have service-related injuries (59). This change can pose new difficulties such as not completing the necessary paperwork to access the benefits available to them [see (10, 60)], which may prohibit Veterans from initiating or continuing mental health care in the post-service period. Additionally, Veteran samples noted a lack of military cultural competence and/or unfamiliarity with treating mental health conditions among providers. Veterans represent a distinct cultural group which may have different health care needs relative to non-Veterans. Tam-Seto et al. (61) notes that a lack of cultural awareness, sensitivity, knowledge, and skills required to meet the unique mental health needs of Veterans can diminish the quality of the therapeutic relationship and impact wellbeing. This finding highlights the importance of the therapeutic alliance in this group [see (62)]. A lack of service preferences (e.g., requests for a female mental health service provider or individual therapy vs. group therapy) was also identified as a prominent barrier by Veterans. Distinct from barriers which impede access to services, service preferences may be equally as important in determining Veteran engagement (63).

Findings also revealed that when these barriers are rectified, these themes can function as significant facilitators. For example, while difficulties navigating the system were the most common barriers, support navigating the system was the most common facilitator. In this sample, Veterans noted the importance of receiving assistance from fellow Veterans, health care providers, and staff members. Similarly, service availability and having health care providers with military cultural competence were other prominent facilitators of receiving mental health care. Indeed, having a variety of mental health services and treatment modalities available is important in addressing individual differences in needs [especially among minority groups such as women Veterans; (49)]. Further, access to providers educated in the unique occupational stressors that are associated with a military career can help to facilitate the transition to the civilian health care system and ensures access to relevant resources (61, 64).

Families of Veterans identified having difficulty navigating the system as the most common systemic barrier to care. Schwartz et al. (9) found that family members of Veterans are often unaware of the formal mental health resources available to them and are unclear on the administrative processes required to access these supports. Service availability was the most reported systemic facilitator; however, the representation of samples is too small to reliably interpret.

#### Interpersonal/intrapersonal

Negative pre-existing attitudes and beliefs were reported among many of the Veteran samples in the review. One review exploring the association between mental health beliefs and service use in military populations found that personal beliefs (about mental health, including stigma) are an important predictor of mental health service use [see (65)]. Veterans also frequently reported gaps in mental health knowledge related to mental health symptoms and the potential treatments available to address them [see (66)]. Increasing mental health knowledge may increase help-seeking and service use by minimizing the impact of other barriers. For example, a study of female Veterans in the U.S. found that increasing mental health knowledge reduced stigma and, thus, indirectly increased mental health treatment-seeking behaviours (67). Veterans also reported a lack of trust in the system, including but not limited to federally operated Veterans Affairs health organizations. One qualitative study found that lack of trust in the U.S. Veterans Affairs (VA) health care system acted as a significant barrier to mental health care-seeking, because of concerns it would be “nonresponsive, ineffective, and uncaring” [see (11)]. Concerns over confidentiality or the ability to provide high-quality services in a timely manner also contribute to a lack of trust in Veteran health organizations (22). Relatedly, a lack of trust in their mental health care provider was also reported as a barrier among some samples. Consistent with the previous themes, mental health knowledge was identified as the most common facilitator among the Veteran samples. This aligns with previous research suggesting that increased mental health knowledge is inversely associated with negative attitudes towards mental health and mental health treatment (68). As in the Veteran samples, gaps in mental health knowledge were the most commonly reported intra-/interpersonal barrier among Veteran family samples while having mental health knowledge was the most frequently reported facilitator. Family members who are more aware of the impact that living with a Veteran experiencing mental health issues can have on their own mental health may be more inclined to seek mental health treatment themselves.

#### Logistical

Transportation challenges were identified as the most prominent logistical barrier in Veteran samples. This encompasses the distance to the closest mental health care provider (i.e., too far to travel) or a lack of a reliable mode of transportation to reach these services. This may be a particular concern for Veterans residing in rural areas, where there are fewer mental health care providers and resources available (11). Lifestyle disruptions, such as needing to request time off work or finding a childcare provider to attend treatment (69), were also reported as barriers to mental health care in this review. It is important to note that these barriers may carry additional costs that render mental health care unaffordable, particularly for Veterans who may not have additional benefits or health care coverage. Convenience was the most reported logistical facilitator in this sample. Convenience may encompass living near mental health services, accessing services remotely, or being able to receive mental health services in the same location as physical health services. Previous research indicates that logistical barriers are frequently reported barriers to health care among military spouses (70). Among the samples of Veteran families included in this review, lifestyle disruptions–such as needing to take time off work or finding childcare–was the most reported barrier. Indeed, Maguire et al. (16), notes that care coordination can be particularly challenging among Veteran families with complex mental health needs (i.e., those impacting more than one domain of functioning), as may be the case during the post-service period. Logistical facilitators to mental health care were not widely commented on by the Veteran families included in the review.

#### Social environmental

The stereotypical military characteristics of stoicism and self-reliance were the most commonly reported social environmental barriers to seeking mental health care among the Veteran samples included in this review. One study exploring barriers to mental health care among U.S. Veterans found that self-reliance and stoicism were the most common attitudinal barriers to mental health care, with many Veterans reported enduring their mental health systems without complaint until the need for mental health treatment became undeniable (11). Indeed, another study found that Veterans delayed seeking mental health care for almost twelve years following their release from the military [see (71)]. Concerns about the potential consequences mental health care-seeking might have on their military careers were also identified as a common barrier. Some of these concerns may include being treated differently by military leadership (72) or adverse military career implications (73). Additionally, gender stereotypes were identified as a barrier to mental health care in some studies. Previous research shows that men and women Veterans experience different barriers to mental health care, with women being more likely to indicate that their gender itself, as well as gender-based discrimination experienced during their military service, act as barriers to seeking mental health services (74). Female Veterans may also feel uncomfortable in male- dominated health service environments, such as VA mental health clinics (75).

Social support was the only social environmental facilitator cited in the Veteran samples included in this review. Positive social support (from partners, civilian communities, peers, etc.) has been identified as a motivator for seeking mental health care in a number of studies involving military Veterans [e.g., (11, 25, 55)]. Further, research suggests that mentor/mentee-like relationships with other Veterans who have experienced and successfully sought help for a mental health concern may be a particularly valuable source of social support for Veterans contemplating mental health treatment initiation (11).

Few Veteran family members identified any social environmental barriers to mental health care: only lack of social support was identified in one of the Veteran family samples as a barrier to mental health care. Previous research has identified lack of social support as a generalized barrier to mental health care (16); however, it may be possible that this is significantly less of a concern for Veteran family members than it appears to be for Veterans themselves.

Positive social support was the only social environmental facilitator of mental health care identified in the Veteran family samples. Recent research suggests that family involvement in treatment for military-related posttraumatic stress disorder is, for some, motivated by social relationships [e.g., improving family life; protecting familial relationships; (52)]. This may be a unique form of social support that motivates Veteran family members to engage in mental health care.

### Mapping barrier and facilitator themes onto the Theoretical Domains Framework

The barriers and facilitators to mental health care identified in this review mapped on to 6 out of 14 TDF constructs: knowledge, social/professional role and identity, beliefs about capabilities, beliefs about consequences, environmental context and resources, and social influences (see Table 4).

Most of the barriers identified in this review reflected the environmental context and resources, and social influences related to mental health care experiences. According to the TDF Behaviour Change Wheel [see (21)], environmental context and resources and social influences jointly contribute to opportunity. Barriers within these domains limit opportunity and influence both physical and social sources of behaviour. In the context of this review, Veterans and Veteran families reported how their engagement in mental health care services are limited by numerous barriers affecting access and availability (e.g., cost, service preferences), and the social contexts they are embedded within (e.g., military culture of stoicism, gender stereotypes). Per the TDF these barriers are inhibiting both physical and social components of treatment-seeking behaviours. These findings highlight specific areas (i.e., enhancing opportunity via environmental and social strategies) for policy recommendations or interventions aimed at engendering behavioural change related to help-seeking in these populations.

Notably, the environmental context and resources domain also emerged as an important facilitator of mental health care experiences. As previously noted, these facilitators often reflect the inverse of barriers (e.g., service availability vs. lack of service availability), highlighting the importance of rectifying prominent barriers to optimize experience and outcomes. Further, this finding serves to help refine our examination of gaps in services and supports with respect to the TDF. While environmental factors detract from opportunity, most facilitators also fell into this category, while facilitators with respect to social influences were very limited (i.e., social support only). Taken together, it may be that social influences are disproportionately detracting from opportunity relative to environmental factors; however, the representation of facilitators was relatively limited, and this review did not determine the magnitude of the effect of these barriers and facilitators beyond reported frequency. The knowledge domain also emerged as an important factor in facilitating experiences of Veterans and Veteran families' experiences with mental health care services. With respect to the TDF Behaviour Change Wheel, knowledge (e.g., mental health knowledge, and support navigating the system) bolsters capability and positively influences psychological sources of help-seeking behaviours.

### Implications for research and policy

The findings of this review of qualitative and quantitative research align well with a rapid review conducted by Randles & Finnegan (26), as well as Hitch et al.'s (25) systematic review of quantitative research exploring barriers and facilitators to health care-seeking. Despite differences in the types of studies included across all three reviews, the consistency of findings increases confidence in the reliability and accuracy of our findings.

The heterogeneity in reported experiences observed across barriers and facilitators included in this review suggests that perhaps access to the system is not standardized or is based on other factors, such as reason for release from the military or familiarity with the system. Additionally, variability in structure, availability, and cost of mental health services between and within countries (e.g., state to state) may account for some of the variability with respect to thematic valence. However, these variations (i.e., experiencing a systemic factor as a barrier or facilitator) can be used to identify opportunities to create equitable policies that increase access to mental health services. The use of objective measures of barriers to mental health care (e.g., wait time from referral to support) may help to disentangle some of the heterogeneity observed in this review.

Findings of this review may also be used to inform relevant policy recommendations at the federal and provincial/community level. Concerted efforts to address the systemic, logistical, social environmental, and intra-/interpersonal barriers to mental health care should occur conjointly in order to maximize their benefit. For example, this might include building upon recent efforts by the CAF to increase mental health awareness and reduce stigma. Reducing stigma around service-related mental health concerns may promote help-seeking behaviours in military (and subsequently Veteran) populations. Federal agencies should aim to ensure the availability of culturally competent providers within their networks while also exploring avenues to decrease logistical and demand barriers to mental health care, such as providing childcare support and offering women-only hours in their mental health clinics. Nevertheless, access to mental health care for Veterans and Veteran families cannot be solved through addressing one type of barrier alone. For example, efforts to decrease stigma will not get more Veterans into treatment if supply issues are not addressed or the system remains difficult to navigate.

Similar steps could be taken at the provincial health care level. The civilian health care system is not always well equipped to treat the mental health needs of Veterans, particularly in areas without a large military or Veteran population. Efforts to identify the root cause(s) of insufficient military cultural competency, such as lack of training, could help address the paucity of community-based mental health providers with military cultural competence, while targeted recruitment efforts could be used to increase the number of mental health care providers in rural regions.

### Limitations and strengths

The findings of this review should be interpreted in consideration of a few limitations. First, we were unable to conduct subgroup analyses. As such, the overall findings of this review disproportionately reflect experiences of mental health care for an American Veteran population via qualitative experiential accounts relative to mixed methods designs and do not adequately reflect the experiences of Veterans from other Five Eyes nations, and Veterans with intersectional identities. While these countries share important similarities, the inclusion of samples from Australia and New Zealand in addition to a more robust representation of Canadian and UK samples in this review may have allowed us to capture meaningful trends in access across various types of health care delivery systems (e.g., public vs. private), which would have provided important contextual nuance with respect to these experiential findings especially as they related to barriers associated with cost. Further efforts to disentangle whether the systemic barriers and facilitators to mental health care vary across nations and health care systems would help solidify our understanding of the specific barriers and facilitators faced by Canadian Veterans and families. Second, most studies did not provide information about the amount of time between data collection and end of participants' military service. The military to civilian transition period, which can be a period of increased need for mental health services, may have different barriers or facilitators to mental health care than in the years following this transitional period. Finally, our review contained only a few samples of Veteran families. This review adds to a growing body of literature attempting to elucidate and contextualize experiences of mental health care for Veteran families and calling for additional research within this population.

This review also had several strengths. First, this review provides a much-needed synthesis of literature in this area and summarizes available information on barriers and facilitators to mental health care for both Veterans and Veteran families. Importantly, this review highlighted that when Veteran health organizations address barriers to mental health care, these barriers became facilitators, enabling access to mental health services. Second, the quality of the studies included in the review were adequate, as measured by the MMAT, indicating that information included in this review was collected and analyzed with rigour. Finally, the findings of this review, in conjunction with the context provided by the TDF, highlight opportunities for future research, intervention approaches, and policy changes.

### Considerations for future directions

Future studies in this field should aim to pinpoint specific behavioural determinants of health-seeking via primary data collection with Veterans and Veteran families. Using a structured framework such as the TDF would provide a more comprehensive context-specific understanding, and capture data that is reflective of current policies and community attitudes. Second, studies should implement conjoint analyses to empirically examine the relative importance of specific barriers and facilitators to treatment-seeking. Similarly, given that these results are based on mostly qualitative studies, future studies should seek to understand how themes related to help-seeking and service use among these populations are represented in both qualitative and quantitative data. For example, if themes related to stigma are more commonly captured in qualitative research, then it becomes important to identify the best way to capture these themes in a quantitative capacity as well. Third, studies should aim to capture the unique barriers and facilitators to mental health care for Veterans' and their families in a Canadian context. Relatedly, future studies should attempt to better understand help-seeking behaviours with respect to individual differences and across social identities (e.g., gender, age, sexual orientation, etc.). Finally, families represent a broad group, and future research should consider the barriers and facilitators for different kinds of family members and dynamics (e.g., spouses, children).

## Conclusion

This review identified several barriers and facilitators to mental health care for Veterans and Veteran families. While systemic barriers, such as difficulty navigating systems were commonly reported by Veterans and Veteran families, these factors were also identified as facilitators to mental health care when addressed. These findings highlight the heterogeneity in Veterans' and Veteran families' experiences with mental health care-seeking, and the need to understand the effects of barriers on help-seeking behaviours and experiences to implement the appropriate modifications to remove them. In doing so, Veteran health and well-being organizations can provide relevant and accessible mental health care and, subsequently, improve mental health outcomes for Veterans and Veteran families.

## Data Availability

The original contributions presented in the study are included in the article/Supplementary Material, further inquiries can be directed to the corresponding author.
