# Supplementary material for: A rapid review of the barriers and facilitators of mental health service access among Veterans and their families
Source: Front Health Serv. 2024 Jul 22;4:1426202. doi: 10.3389/frhs.2024.1426202 (PMC11298457; doi:10.3389/frhs.2024.1426202)
Supplement: Supplementary file 2 [file Table1.docx]

# Supplementary Material

**Table of Contents**

[Supplementary Material 1](#_Toc164765356)

[String Terms 2](#_Toc164765357)

[Raw Data of Included Studies 4](#_Toc164765358)

[References for Included Articles 5](#_Toc164765359)

##

## String Terms

N.B. *n* refers to number of citations.

*Search Date:* December 18, 2023

**Scopus (n = 3330)**

Search within: Article title

( ( TITLE ( ( resources OR "mental health care" OR programs OR "mental health service use" OR "mental health services use" OR "mental healthcare" OR "mental health support" OR "mental health treatment" OR "mental health use" OR "psychiatr* service use" OR "utili*ation" OR "help-seeking" OR "mental health" ) ) AND TITLE ( ( ( military OR "armed forces" OR soldier OR rcmp OR veteran* ) W/3 retir* ) ) ) ) OR ( ( TITLE ( ( resources OR "mental health care" OR programs OR "mental health service use" OR "mental health services use" OR "mental healthcare" OR "mental health support" OR "mental health treatment" OR "mental health use" OR "psychiatr* service use" OR "utili*ation" OR "help-seeking" OR "mental health" ) ) AND TITLE ( ( ( military OR "armed forces" OR soldier OR rcmp OR veteran* ) W/3 transi* ) ) ) ) OR ( ( TITLE ( ( resources OR "mental health care" OR programs OR "mental health service use" OR "mental health services use" OR "mental healthcare" OR "mental health support" OR "mental health treatment" OR "mental health use" OR "psychiatr* service use" OR "utili*ation" OR "help-seeking" OR "mental health" ) ) AND TITLE ( ( military OR "armed forces" OR soldier OR rcmp OR veteran* ) ) ) ) AND PUBYEAR > 2012 AND PUBYEAR < 2025

**PsycINFO (OVID; n = 1243)**

1. (resources or "mental health care" or programs or "mental health service use" or "mental health services use" or "mental healthcare" or "mental health support" or "mental health treatment" or "mental health use" or "psychiatr* service use" or "utili*ation" or "help-seeking" or "mental health").m.titl

2. (military or “armed forces” or soldier or rcmp or veteran*).m.titl.

3. (military or “armed forces” or soldier or rcmp or veteran* adj3 transiti*).m.titl.

4. (military or “armed forces” or soldier or rcmp or veteran* adj3 retir*).m.titl.

5. (1 and 2) or (1 and 3) or (1 and 4)

6. limit 5 to yr="2013-Current"

**Medline (OVID; n = 1568)**

1. (resources or "mental health care" or programs or "mental health service use" or "mental health services use" or "mental healthcare" or "mental health support" or "mental health treatment" or "mental health use" or "psychiatr* service use" or "utili*ation" or "help-seeking" or "mental health").m.titl

2. (military or “armed forces” or soldier or rcmp or veteran*).m.titl.

3. (military or “armed forces” or soldier or rcmp or veteran* adj3 transiti*).m.titl.

4. (military or “armed forces” or soldier or rcmp or veteran* adj3 retir*).m.titl.

5. (1 and 2) or (1 and 3) or (1 and 4)

6. limit 5 to yr="2013-Current"

##

## Raw Data of Included Studies

See excel file labeled “Supplementary Material - Raw Data.”

## References for Included Articles

Albright, D. L., McCormick, W. H., Carroll, T. D., Currier, J. M., Thomas, K. H., Hamner, K., Slagel, B. A., Womack, B., Sims, B. M., & Deiss, J. (2018). Barriers and resources for Veterans’ post-military transitioning in South Alabama: A qualitative analysis. *Traumatology*, *24*(3), 236–245. https://doi.org/10.1037/trm0000147

AlMasarweh, L., & Ward, C. (2016). Barriers to health care access and utilization: A study of Native American women Veterans in two Montana reservations. *Research in the Sociology of Health Care*, *34*, 33–60. https://doi.org/10.1108/s0275-495920160000034003

Beks, T. A., & Cairns, S. L. (2020). Factors that influence mental health service use among female partners of posttraumatic stress disorder-affected Veterans. *Traumatology*. https://doi.org/10.1037/trm0000240

Bishop-Deaton, D. (2019). Veteran anger dysregulation: A phenomenological analysis of help-seeking through social media (Publication No. 13861273) [Doctoral dissertation, Walden University]. ProQuest Dissertations Publishing.

Bovin, M. J., Miller, C. J., Koenig, C. J., Lipschitz, J. M., Zamora, K. A., Wright, P. B., Pyne, J. M., & Burgess, J. F. (2019). Veterans’ experiences initiating VA-based mental health care. *Psychological Services*, *16*(4), 612–620. https://doi.org/10.1037/ser0000233

Brooks, E., Dailey, N. K., Bair, B. D., & Shore, J. H. (2016). Listening to the patient: Women Veterans’ insights about health care needs, access, and quality in rural areas. *Military Medicine*, *181*(9), 976–981. https://doi.org/10.7205/milmed-d-15-00367

Brown, N. (2017). *Telemental health versus face-to-face treatment: An examination of Operation Enduring Freedom and Operation Iraqi Freedom Veterans' preferences for mental health services* (Publication No. 10606703) [Doctoral dissertation, University of Missouri-Saint Louis]. ProQuest Dissertations Publishing.

Brunner, J., Schweizer, C. A., Canelo, I. A., Leung, L. B., Strauss, J. L., & Yano, E. M. (2019). Timely access to mental health care among women Veterans. *Psychological Services*, *16*(3), 498–503. https://doi.org/10.1037/ser0000226

Campbell, G. M., Williamson, V., & Murphy, D. (2023). “A hidden community”: The experiences of help-seeking and receiving mental health treatment in U.K. women Veterans. A qualitative study. *Armed Forces & Society*. https://doi.org/10.1177/0095327x231182140

Castro, F., AhnAllen, C. G., Wiltsey-Stirman, S., Lester-Williams, K., Klunk-Gillis, J., Dick, A. M., & Resick, P. A. (2015). African American and European American Veterans’ perspectives on receiving mental health treatment. *Psychological Services*, *12*(3), 330–338. https://doi.org/10.1037/a0038702

Chen, P. V., Helm, A., Fletcher, T., Wassef, M., Hogan, J., Amspoker, A., Cloitre, M., & Lindsay, J. (2021). Seeing the value of video: A qualitative study on patient preference for using video in a Veteran Affairs telemental health program evaluation. *Telemedicine Reports*, *2*(1), 156–162. <https://doi.org/10.1089/tmr.2021.0005>

Cheney, A. M., Koenig, C. J., Miller, C. J., Zamora, K., Wright, P., Stanley, R., Fortney, J., Burgess, J. F., & Pyne, J. M. (2018). Veteran-centered barriers to VA mental healthcare services use. *BMC Health Services Research*, *18*(1). https://doi.org/10.1186/s12913-018-3346-9

Clary, K. L., Pena, S., & Smith, D. C. (2021). Masculinity and stigma among emerging adult military members and Veterans: Implications for encouraging help-seeking. *Current Psychology*, *42*, 4422–4438. https://doi.org/10.1007/s12144-021-01768-7

Cornish, M. A., Thys, A., Vogel, D. L., & Wade, N. G. (2014). Post-deployment difficulties and help seeking barriers among military Veterans: Insights and intervention strategies. *Professional Psychology: Research and Practice*, *45*(6), 405–409. https://doi.org/10.1037/a0037986

Daly, D. (2020). *Perceptions of substance use and service utilization among veterans experiencing homelessness* (Publication No. 27956092) [Doctoral dissertation, University of the Sciences in Philadelphia]. ProQuest Dissertations Publishing.

DiNapoli, E. A., Cinna, C., Whiteman, K. L., Fox, L., Appelt, C. J., & Kasckow, J. (2016). Mental health treatment preferences and challenges of living with multimorbidity from the Veteran perspective. *International Journal of Geriatric Psychiatry*, *31*(10), 1097–1104. https://doi.org/10.1002/gps.4550

Drummelsmith, J. (2020). *Understanding the mental health needs and perceptions of incarcerated Canadian veterans*(Publication No. 13898583) [Doctoral dissertation, The Chicago School of Professional Psychology]. ProQuest Dissertations Publishing.

Farrand, P., Jeffs, A., Bloomfield, T., Greenberg, N., Watkins, E., & Mullan, E. (2018). Mental health service acceptability for the armed forces Veteran community. *Occupational Medicine*, *68*(6), 391–398. https://doi.org/10.1093/occmed/kqy086

Fischer, E. P., McSweeney, J. C., Wright, P., Cheney, A., Curran, G. M., Henderson, K., & Fortney, J. C. (2016). Overcoming barriers to sustained engagement in mental health care: Perspectives of rural Veterans and providers. *The Journal of Rural Health*, *32*(4), 429–438. https://doi.org/10.1111/jrh.12203

Garcia, H. A., Finley, E. P., Ketchum, N., Jakupcak, M., Dassori, A., & Reyes, S. C. (2014). A survey of perceived barriers and attitudes toward mental health care among OEF/OIF Veterans at VA outpatient mental health clinics. *Military Medicine*, *179*(3), 273–278. https://doi.org/10.7205/milmed-d-13-00076

Gayles, A. (2021). *Understanding the mental healthcare needs of female Veterans and access to mental health care services within a Veterans health care facility: A phenomenological case study* (Publication No. 28776688) [Doctoral dissertation, Baylor University]. ProQuest Dissertations Publishing.

Godier-McBard, L. R., Cable, G., Wood, A. D., & Fossey, M. (2021). Gender differences in barriers to mental healthcare for UK military veterans: A preliminary investigation. *BMJ Military Health*, *168*(1). https://doi.org/10.1136/bmjmilitary-2020-001754

Hahn, C. K., Turchik, J., & Kimerling, R. (2020). A latent class analysis of mental health beliefs related to military sexual trauma. *Journal of Traumatic Stress*, *34*. https://doi.org/10.1002/jts.22585

Harris, M. P. J., Palmedo, P. C., & Fleary, S. A. (2022). “What gets people in the door”: An integrative model of student veteran mental health service use and opportunities for communication. *Journal of American College Health*, 1–11. https://doi.org/10.1080/07448481.2022.2129977

Hilgeman, M. M., Cramer, Dr. R. J., Hoch, M. C., Collins, A. N., Zabelski, S., & Heebner, N. R. (2022). A pilot study comparing two measures of perceived health services access among military Veterans with musculoskeletal injuries and mental health conditions. *Military Medicine*, *188*(7/8:e2363). https://doi.org/10.1093/milmed/usac072

Houle, S. A., Pollard, C., Jetly, R., & Ashbaugh, A. R. (2022). Barriers and facilitators of help seeking among morally injured Canadian Armed Forces Veterans and service members: A qualitative analysis. *Journal of Military, Veteran and Family Health*, *8*(3), 58–71. https://doi.org/10.3138/jmvfh-2021-0093

Ingelse, K., & Messecar, D. (2016). Rural women Veterans’ use and perception of mental health services. *Archives of Psychiatric Nursing*, *30*(2), 244–248. https://doi.org/10.1016/j.apnu.2015.11.008

Kimerling, R., Pavao, J., Greene, L., Karpenko, J., Rodriguez, A., Saweikis, M., & Washington, D. L. (2015). Access to mental health care among women Veterans. *Medical Care*, *53*, S97–S104. https://doi.org/10.1097/mlr.0000000000000272

Knight, M. K. (2019). *Barrier to mental health care for combat Veterans: A qualitative study* (Publication No. 22619322) [Doctoral dissertation, Adler University]. ProQuest Dissertations Publishing.

Koblinsky, S. A., Schroeder, A. L., & Leslie, L. A. (2016). “Give us respect, support and understanding”: Women Veterans of Iraq and Afghanistan recommend strategies for improving their mental health care. *Social Work in Mental Health*, *15*(2), 121–142. https://doi.org/10.1080/15332985.2016.1186134

Kochel, T. L. (2020). *Underutilization of behavioral health resources amongst military Veterans*. [Doctoral dissertation, Northcentral University].

Koenig, C. J., Abraham, T., Zamora, K. A., Hill, C., Kelly, P. A., Uddo, M., Hamilton, M., Pyne, J. M., & Seal, K. H. (2016). Pre-implementation strategies to adapt and implement a Veteran peer coaching intervention to improve mental health treatment engagement among rural Veterans. *The Journal of Rural Health*, *32*(4), 418–428. https://doi.org/10.1111/jrh.12201

Kulesza, M., Pedersen, E. R., Corrigan, P. W., & Marshall, G. N. (2015). Help-seeking stigma and mental health treatment seeking among young adult Veterans. *Military Behavioral Health*, *3*(4), 230–239. https://doi.org/10.1080/21635781.2015.1055866

Lara-Smith, T. A. (2022). *A phenomenological study on the perceptions of Operation Enduring Freedom and Operation Iraq Freedom or Operation New Dawn Service members with PTSD and spouses on the mental health care provided by the US military* (Publication No. 28863603) [Doctoral dissertation, Northcentral University]. ProQuest Dissertations Publishing.

Mellotte, H., Murphy, D., Rafferty, L., & Greenberg, N. (2017). Pathways into mental health care for UK veterans: a qualitative study. *European Journal of Psychotraumatology*, *8*(1389207). https://doi.org/10.1080/20008198.2017.1389207

Moore, R. C., Marquine, M. J., Straus, E., Depp, C. A., Moore, D., Schiehser, D. M., Richtand, N. M., Jeste, D. V., & Eyler, L. T. (2017). Predictors and barriers to mental health treatment utilization among older Veterans living with HIV. *Primary Care Companion to the Journal of Clinical Psychiatry*, *19*(01). https://doi.org/10.4088/pcc.16m02059

Murphy, D., Palmer, E., & Busuttil, W. (2016). Mental health difficulties and help-seeking beliefs within a sample of female partners of UK Veterans diagnosed with post-traumatic stress disorder. *Journal of Clinical Medicine*, *5*(68). https://doi.org/10.3390/jcm5080068

Murray-Swank, N. A., Dausch, B. M., & Ehrnstrom, C. (2018). The mental health status and barriers to seeking care in rural women Veterans. *Journal of Rural Mental Health*, *42*(2), 102–115. https://doi.org/10.1037/rmh0000095

Nichter, B., Hill, M., Norman, S., Haller, M., & Pietrzak, R. H. (2020). Mental health treatment utilization among U.S. military veterans with suicidal ideation: Results from the national health and resilience in Veterans study. *Journal of Psychiatric Research*, *130*, 61–67. https://doi.org/10.1016/j.jpsychires.2020.07.004

Opelt, B. L. (2021). *Military student Veterans’ mental health help-seeking* (Publication No. 28548728) [Doctoral dissertation, New Mexico State University]. ProQuest Dissertations Publishing.

Perry, L. K. (2023). *Perceptions of female Veterans regarding mental health services in the Veterans Administration System*(Publication No. 30001111) [Doctoral dissertation, Walden University]. ProQuest Dissertations Publishing

Porter O, K. (2013). *Hear my battle-cry: Understanding troop needs and views of military mental health as told by the troops.* [Doctoral Dissertation, The Chicago School of Professional Psychology]. ProQuest Dissertations Publishing.

Possemato, K., Wray, L. O., Johnson, E., Webster, B., & Beehler, G. P. (2018). Facilitators and barriers to seeking mental health care among primary care Veterans with posttraumatic stress disorder. *Journal of Traumatic Stress*, *31*(5), 742–752. https://doi.org/10.1002/jts.22327

Pyne, J. M., Kelly, P. A., Fischer, E. P., Miller, C. J., Wright, P., Zamora, K., Koenig, C. J., Stanley, R., Seal, K., & Fortney, J. C. (2019). Development of a perceived access inventory for community care mental healthcare services for Veterans. *Military Medicine*, *184*(7/8), e301–e308. https://doi.org/10.1093/milmed/usy429

Pyne, J. M., Rabalais, A., & Sullivan, S. (2019). Mental health clinician and community clergy collaboration to address moral injury in Veterans and the role of the Veterans Affairs chaplain. *Journal of Health Care Chaplaincy*, *25*, 1-19. doi: 10.1080/08854726.2018.1474997

Rafferty, L. A., Wessely, S., Stevelink, S. A. M., & Greenberg, N. (2019). The journey to professional mental health support: A qualitative exploration of the barriers and facilitators impacting military Veterans’ engagement with mental health treatment. *European Journal of Psychotraumatology*, *10*(1700613). https://doi.org/10.1080/20008198.2019.1700613

Rosado, J. C. (2017). *Interventions to reduce perceived stigma for mental health treatment in a Veteran population: Do peer contact, psychoeducation, and empathy make a difference?* (Publication No. 10607939) [Doctoral dissertation, Fielding Graduate University]. ProQuest Dissertations Publishing.

Schvey, N. A., Burke, D., Pearlman, A. T., Britt, T. W., Riggs, D. S., Carballo, C., & Stander, V. (2021). Perceived barriers to mental healthcare among spouses of military service members. *Psychological Services*, *19*. https://doi.org/10.1037/ser0000523

Schwartz, K. D., Norris, D., Cramm, H., Tam-Seto, L., & Mahar, A. (2021). Family members of Veterans with mental health problems: seeking, finding, and accessing informal and formal supports during the military-to-civilian transition. *Journal of Military, Veteran and Family Health*, *7*(1), 21–34. https://doi.org/10.3138/jmvfh-2019-0023

Shaine, M. J. D., Cor, D. N., Campbell, A. J., & McAlister, A. L. (2021). Mental health care experiences of trans service members and Veterans: A mixed‐methods study. *Journal of Counseling & Development*, *99*(3), 273–288. http://doi.org/10.1002/jcad.12374

Shaw, J. W. (2018). *The effects of an intervention on Veterans' help-seeking behavior from civilian mental health practitioners*(Publication No. 10748900) [Doctoral dissertation, Trevecca Nazarene University]. ProQuest Dissertations Publishing.

Shepherd-Banigan, M., Shapiro, A., Sheahan, K. L., Ackland, P. E., Meis, L. A., Thompson-Hollands, J., Edelman, D., Calhoun, P. S., Weidenbacher, H., & Van, C. H. (2023). Mental health therapy for veterans with PTSD as a family affair: A qualitative inquiry into how family support and social norms influence Veteran engagement in care. *Psychological Services*, *20*(4). https://doi.org/10.1037/ser0000742

Shepherd-Banigan, M., Shapiro, A., Stechuchak, K. M., Sheahan, K. L., Ackland, P. E., Fritsch, V. A., Bokhour, B. G., Glynn, S. M., Calhoun, P. S., Edelman, D., Weidenbacher, H. J., Eldridge, M. R., & Harold, C. (2023). Exploring the importance of predisposing, enabling, and need factors for promoting Veteran engagement in mental health therapy for post-traumatic stress: A multiple methods study. *BMC Psychiatry*, *23*(1). https://doi.org/10.1186/s12888-023-04840-7

Silvestrini, M., & Chen, J. A. (2022). “It’s a sign of weakness”: Masculinity and help-seeking behaviors among male Veterans accessing posttraumatic stress disorder care. *Psychological Trauma: Theory, Research, Practice, and Policy*, *15*(4). https://doi.org/10.1037/tra0001382

Skellon, N. (2016). *Factors that impact on military personnel and military Veterans accessing mental health services*(Publication No*.* 28782520) [Doctoral dissertation, The University of Liverpool (United Kingdom)]. ProQuest Dissertations Publishing.

True, G., Rigg, K. K., & Butler, A. (2015). Understanding barriers to mental health care for recent war Veterans through photovoice. *Qualitative Health Research*, *25*(10), 1443–1455. https://doi.org/10.1177/1049732314562894

Tsai, J., Mota, N. P., & Pietrzak, R. H. (2015). U.S. female Veterans who do and do not rely on VA health care: Needs and barriers to mental health treatment. *Psychiatric Services*, *66*(11), 1200–1206. https://doi.org/10.1176/appi.ps.201400550

Williams, L. (2015). *Women Veterans' perceptions of mental health outpatient services*. [Doctoral Dissertation, University of California]. ProQuest Dissertations Publishing.

Williams, L. A. (2023). *Factors related to utilization of Veterans Affairs (VA) mental healthcare services by female Veterans with depression* (Publication No. 30422832) [Doctoral dissertation, The Catholic University of America]. ProQuest Dissertations Publishing.

Wray, L. O., Pikoff, E., King, P. R., Hutchison, D., Beehler, G. P., & Maisto, S. A. (2016). Veterans’ mental health beliefs: Facilitators and barriers to primary care-mental health use. *Families, Systems, & Health*, *34*(4), 404–413. https://doi.org/10.1037/fsh0000231
